# Editorial: Role of Non-Coding RNAs in Animals

**DOI:** 10.3390/ani13050805

**Published:** 2023-02-23

**Authors:** Duy Ngoc Do, Prashanth Suravajhala

**Affiliations:** 1 Faculty of Veterinary Medicine, Viet Nam National University of Agriculture, Hanoi 100000, Vietnam; 2 Department of Animal Science and Aquaculture, Dalhousie University, Truro, NS B2N 5E3, Canada; 3 Bioclues.org, Hyderabad 500072, India; 4 Amrita School of Biotechnology, Amrita Vishwa Vidyapeetham, Clappana 690525, India

The importance of non-coding RNAs (ncRNAs), such as microRNAs (miRNA), long non-coding RNAs (lncRNA), and circular RNAs (circRNA), in gene regulation is increasingly being appreciated in many species. Thanks to the next generation of sequencing methods, thousands of ncRNAs have been identified in different livestock species and their functions are undergoing characterization. Clearly, ncRNAs are involved in many gene regulation processes [1] and pathways related to economically important traits in livestock species [2,3,4,5,6]. In addition, ncRNAs are also considered to be potential biomarkers for infectious diseases in different farm animal species [7,8]. This Special Issue contains 13 articles summarizing the diverse roles of ncRNAs in livestock [9,10,11,12,13,14,15,16,17,18,19,20,21]. In particular, we report on different species of animal that have been used to study the functions of ncRNAs. These include chicken [12,15,18], pigs [11,17,21], goats [9], sheep [14,16], cattle [13,19], mice [20], and insects [10].

Exploring the expression of ncRNAs in different biological conditions is an important step in understanding their function. Bo et al. [9] identified 20,269 lincRNAs, including 16,931 novel lncRNAs, expressed in the testes of Yiling goats. The authors also suggested that ENSCHIT00000000777, ENSCHIT00000002069, and ENSCHIT00000005076 were the key lincRNAs in the process of testis development, a fact derived via co-expression analyses. Gan et al. [11] used RNA sequencing to identify 302 miRNAs expressed in pig umbilical venous blood (UVB) and umbilical arterial blood (UAB). As a result, 106 and 22 miRNAs were specifically expressed in the UVB and UAB, respectively. The authors also suggested that miR-423 and miR-122-5p can be used as characteristic miRNAs of UVB and UAB, respectively. Hao et al. [14] performed RNA-Seq to study the roles of lncRNAs in the mammary glands of lactating Small-Tailed Han (STH) ewes and Gansu Alpine Merino (GAM) ewes. The authors identified 1894 lncRNAs that were differentially expressed, among which 31 and 37 lncRNAs were up and downregulated respectively, when comparing STH ewes with GAM ewes. In addition, the authors also found that the development and proliferation of mammary epithelial cells via the enrichment of the target genes, followed by the morphogenesis of the mammary gland, ErbB signaling pathway, and Wnt signaling pathway could be important for mammary gland and milk yield regulation. To explore the function of lncRNAs in pig spleens at different stages of development, Li et al. [17] profiled the systematic characters of mRNA and lncRNA repertoires in three groups of spleens from nine Yorkshire pigs, with three aged seven days, 90 days, and 180 days, respectively. The authors identified 19,647 genes in addition to 219 known and 3219 putative lncRNA transcripts; 1729 genes and 64 lncRNAs therein were found to express differentially. The authors also found that differentially expressed genes and the potential target genes of differentially expressed lncRNAs both performed the crucial roles of up-regulation in immune activation and hematopoiesis, and down-regulation in cell replication and division, in 90- and 180-day-old pigs compared to seven-day-old pigs. 

MiRNAs are the most well-studied molecules in the ncRNAs. Several studies contained within this Special Issue attempt to validate their functions. Guo et al. indicated the potential roles of miR-149-5b in the regulation of bovine adipocyte differentiation [13]. Xu et al. show that, by targeting PTEN, MicroRNA-148a can regulate the proliferation and differentiation of ovine preadipocytes. Qiao et al. provided evidence of the role of miR-F4-C12 50 in the regulation of adipose accumulation, finding that it performed this role by targeting PIK3R1 in castrated male pigs [21]. Wang et al. [20] identified 852 known miRNAs and 179 novel miRNAs in female ICR mice mammary glands at the virgin stage, day 16 of pregnancy, day 12 of lactation, day 1 of forced weaning, and day 3 of forced weaning. The authors also discovered a novel miRNA (novel-mmu-miR424-5p) involved in regulating the expression of the CSN2 gene.

Other studies also validated the function of circRNAs [18,19] and lncRNAs [15]. Regarding the circRNA function, Wang et al. indicated that circEZH2 can sponge miR-378b to regulate milk fat metabolism [19], while Shen et al. showed that circular PPP1R13B can target miR-9-5p to promote chicken skeletal muscle satellite cell proliferation and differentiation [18]. Lastly, via RNA sequencing and the follow-up validation, Jian et al. [15] confirmed that the roles of lncPGC in the regulation of primordial germ cell formation in chickens were mediated by TCF7L2. This Special Issue focused not only on livestock species but also on the roles of lncRNAs in insects. Choudhary et al. [10] provided an updated review of the functions and molecular mechanisms of the mode of action of different insect lncRNAs.

Taken together, these contributions enlighten the research community on contemporary breakthroughs and suggest approaches for performing future functional studies of the regulatory roles of ncRNAs in animals. We anticipate that both expert scientists and readers can benefit from the state-of-the-art studies addressing the roles of ncRNAs contained within this Special Issue.
